# Impact of preexisting digestive problems on the gastrointestinal symptoms of patients with omicron variant of SARS-CoV-2 infection

**DOI:** 10.1371/journal.pone.0312545

**Published:** 2024-10-30

**Authors:** Xinghuang Liu, Bayasgalan Luvsandagva, Dongke Wang, Siran Zhu, Zhiyue Xu, Dan Zhou, Xiaotian Xie, Wei Qian, Xiaohua Hou, Tao Bai

**Affiliations:** Division of Gastroenterology, Union Hospital, Tongji Medical College, Huazhong University of Science and Technology, Wuhan, China; Kyung Hee University School of Medicine, REPUBLIC OF KOREA

## Abstract

**Objective:**

This study focused on the gastrointestinal (GI) symptoms in the omicron variant infection and the related factors based on digestive health.

**Methods:**

A cross-sectional study was conducted on individuals infected with the omicron variant. A structured questionnaire was developed to gather their demographic characteristics, preexisting digestive problems (diseases & symptoms), and clinical manifestations during the infection.

**Results:**

11,484 questionnaires were received from online platforms. 7,929 infected participants were selected based on inclusion and exclusion criteria. Among them, 4,225 (53.3%) were females, and the mean age was 36.0±8.8 years old. In general, the proportion of GI symptoms in the omicron variant infection was 31.4% (62.6% and 25.0% in participants with pre-existing digestive problems and those without, respectively). The participants with pre-existing digestive problems exhibited more severe clinical manifestations during infection compared to those without. Notably, participants with gastrointestinal symptoms during the infection had more severe clinical manifestations, regardless of basic digestive health. Upper, rather than lower GI symptoms were more closely associated with the severity of the clinical manifestations. NSAIDs may increase the occurrence of GI symptoms in participants with a healthy digestive system but not in those with preexisting digestive problems.

**Conclusion:**

Patients infected with the omicron variant may experience more severe clinical symptoms if they have gastrointestinal issues. Digestive health strongly influences the occurrence of gastrointestinal symptoms and the severity of clinical manifestations.

## Introduction

SARS-CoV-2 was proven to be able to infiltrate the digestive system [1, 2]. A meta-analysis conducted in the early stage of the COVID-19 pandemic showed that the overall incidence of gastrointestinal (GI) symptoms was 17.6% [3]. As the virus mutates, the incidence of GI symptoms could also change, however, the published studies reported various results. *Yang*, *et al* found that found that the incidence of diarrhea in Delta (14.58%) was higher than that in the Omicron (3.23%) and Beta (3.36%) [4]. While *Menni*, *et al*. reported no significant differences in diarrhea, abdominal pain, and nausea between delta and omicron variant infections [5]. Whether mutations of the virus enhance its effect on the GI tract or diminish it may remain controversial. Besides, according to numerous published research studies, the overall prevalence of gastrointestinal (GI) symptoms varies widely in the general population, including diarrhea, constipation, and abdominal pain, ranging from 18.9% to 51.9% [6–8]. Therefore, more studies are needed to evaluate the GI symptoms caused by the omicron variant.

To our knowledge, the underlying gastrointestinal diseases were associated with more severe conditions during infection [9]. Maybe, the pre-existing GI problems, including digestive diseases and GI symptoms, may also influence GI symptoms during SARS-CoV-2 infection. Few studies have addressed this influence; therefore, further research is needed to investigate the occurrence of GI symptoms with the omicron variant infection and consider the baseline GI health status.

As for the clinical influence of GI complaints, published studies reported conflicting results. For example, some studies showed that the presence of GI complaints was significantly associated with a longer disease course [10–12]. But other studies showed the presence of diarrhea associated with better outcomes [13, 14]. As a result, under the circumstance of the new variant, Omicron, it is valuable to discuss the related factors and the clinical influence of GI complaints.

Therefore, we conducted a large-scale survey to investigate the prevalence of gastrointestinal symptoms in omicron variant infection. We also explored the associated factors and clinical influence of gastrointestinal symptoms based on the basic gastrointestinal health status.

## Methods

### Participants and design

A cross-sectional study was performed using an online structured questionnaire. The questionnaire was sent in electronic form to the non-selectively general public in China through WeChat (https://www.wechat.com/en/) from Mar 5st to 23rd, 2023. We included adult participants (age ≥ 18 years) who suffered from the SARS-CoV-2 infection with etiology diagnosis (RT-PCR/antigen test) during the epidemic wave of the omicron variant [15, 16]. Pregnant and lactating women were excluded. This project has been approved by the Ethics Committee of Union Hospital, Tongji Medical College.

### Questionnaire design

General characteristics, including age, sex, height, weight, smoking habits, alcohol consumption, medication use for the infection (especially nonsteroidal anti-inflammatory drugs (NSAIDs), which are widely used to reduce fever after infection), and chronic comorbidities were collected. Preexisting digestive illness was evaluated by collecting the previously gastrointestinal diseases (based on the history of previous gastrointestinal diagnosis and/or endoscopic findings) as well as the course and the frequency of the pre-existing GI symptoms, including dysphagia, early satiety, postprandial fullness, nausea, vomiting, belching, abdominal pain, constipation, diarrhea, abdominal distension/bloating, and other (such as fecal incontinence, rectal pain, and ruminant).

Participants were asked about the existence and severity (mild, moderate, and severe) of the above-mentioned GI symptoms during the infection. Other clinical manifestations, such as the respiratory symptoms, the severity of fever, whether having CT (computerized tomography) confirmed pneumonia, whether they need to take medication and/or to stay in the hospital, and the course of the illness, were collected. We also asked the patients about their COVID-19 vaccination and the COVID-19 infection history.

### Preexisting digestive problems and comorbidities

We were planning to investigate the relationship between different types of digestive diseases and post-infection gastrointestinal (GI) symptoms. However, due to the small number of participants with each specific digestive disease in our survey population (as indicated in S1 Fig, a total of 300 participants for all digestive diseases), conducting separate analyses for each disease proved to be unfeasible. The participant’s self-reported previous diagnosis and the endoscopic findings provided were referred to assess their organic digestive diseases. Of the 300 participants with preexisting digestive diseases, 289 underwent gastroscopy or colonoscopy, revealing consistent endoscopic findings.

In our study, we defined organic chronic digestive diseases (including Barrett’s esophagus, chronic gastritis, inflammatory bowel disease (IBD), unhealed peptic ulcer, chronic pancreatitis, liver cirrhosis, history of surgery to alter the structure of the digestive system (except appendectomy) and malignant tumor) as pre-existing digestive diseases. Asymptomatic hepatitis virus carriers, Helicobacter pylori infections and fatty liver are not considered to have pre-existing digestive diseases. Some participants with self-reported functional diseases (such as irritable bowel disease) will be designated as having previous GI symptoms based on their self-reported symptom profile. Preexisting symptoms were defined as frequently reoccurring (more than once per month) GI symptoms for at least three months before the infection.

As for chronic comorbidities, in the questionnaire, we asked participants about their hypertension, diabetes, chronic heart disease, chronic immune diseases, chronic skin diseases, chronic lung diseases, cerebrovascular disease, tumors, etc. Initially, our analysis planned to exclude participants with severe comorbidities such as chronic obstructive pulmonary disease, COPD, malignancy, heart failure, systemic lupus erythematosus, and stroke. In the questionnaires received, the number of participants with severe comorbidities was very small (data was not shown) and were excluded in the first step of exclusion (exclusion of participants with an unclear diagnosis of infection). Therefore, in our study, the comorbidities included diabetes mellitus, hypertension, coronary artery disease, hyperuricemia, gout, chronic skin diseases and rheumatoid arthritis, hyperthyroidism, and hypothyroidism.

### Statistical analysis

The continuous variables were described with mean ± standard deviation. Categorical variables were presented as numbers and proportions. Comparisons between groups were conducted with the t-test (continuous variables with Normal distribution), Mann-Whitney U test (continuous variables without Normal distribution), or χ^2^ test (categorical variables) [17].

The propensity score for matching was estimated using a logistic regression model with the study data. The calculated propensity scores were then used for matching the groups using the nearest matching approach within a caliper distance of 0.02 of the standard deviation. The related factors of GI symptoms were estimated using Kendall’s tau-b correlation analysis. The independent factors of GI symptoms were analyzed by the logistic regression model. For all calculations, p-values <0.05 were considered statistically significant. The statistical analysis was performed using IBM SPSS Statistics 26.0 software.

## Results

**Fig 1:**
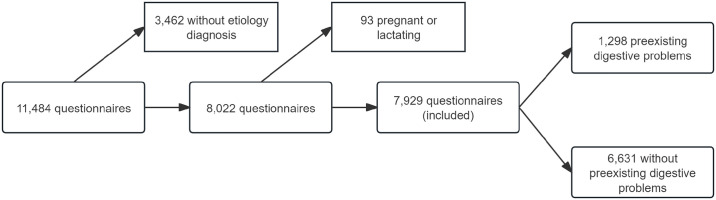
Flow chart of sample screening.

In total, 11,484 participants were submitting the questionnaires. After excluding 3,462 participants without an etiological diagnosis of COVID-19 and 93 pregnant or lactating women, 7,929 records were finally included (Fig 1). Among them, 1,298 participants were considered to have preexisting digestive problems.

### General characteristics of the included participants

Among the 7,929 participants, 4,225 (53.3%) were females (Table 1). The mean age was 36.0±8.8 years old. Compared to participants without preexisting digestive problems, those with preexisting digestive problems were older and had a higher rate of smoking habits, alcohol consumption, and NSAID usage. Additionally, they had a higher incidence of prior infections and a greater number of comorbidities. There were no significant differences in terms of body mass index (BMI) or COVID-19 vaccination history.

**Table 1:** The general characteristics and clinical manifestations of participants based on basic digestive health.

	Items	Preexisting digestive problems	No preexisting digestive problems	P-value	No preexisting digestive problems (matched)	P-value§
		N = 1,298	N = 6,631		N = 2,519	
**General characteristics**	**Sex (female)**	669 (51.5%)	3556 (53.6%)	0.168	1280 (50.8%)	0.988
	**Age (years old)**	36.9±9.8	35.8±8.6	0.001*	36.3±9.1	0.070
	**BMI (kg/m** ^**2**^ **)**	23.6±3.8	23.3±3.6	0.110	23.5±3.7	0.505
	**Having smoking habit**	316 (24.4%)	1254 (18.9%)	0.000*	569 (22.6%)	0.779
	**Alcohol consumption**	894 (68.9%)	4006 (60.4%)	0.000*	1717 (68.2%)	0.908
	**Prior COVID-19 infection**	67 (5.2%)	117 (1.8%)	0.000*	82 (3.3%)	0.403
	**Number of COVID-19 vaccinations**			0.502		0.102
	None	45 (3.5%)	195 (2.9%)		59 (2.3%)	
	1–2	351 (27.0%)	1750 (26.4%)		678 (26.9%)	
	≥3	902 (69.5%)	4686 (70.7%)		1782 (70.7%)	
	**Use of NSAIDs**	961 (74.0%)	5083 (76.7%)	0.047*	1920 (76.2%)	0.480
	**Comorbidities**	242 (18.6%)	557 (8.4%)	0.000*	413 (16.4%)	0.632
**GI complaints**	**Dysphagia**	123 (9.5%)	138 (2.1%)	0.000*	57 (2.3%)	0.000*
	**Early satiety**	118 (9.1%)	208 (3.1%)	0.000*	88 (3.5%)	0.000*
	**Postprandial fullness**	216 (16.6%)	287 (4.3%)	0.000*	122 (4.8%)	0.000*
	**Nausea**	267 (20.6%)	667 (10.1%)	0.000*	263 (10.4%)	0.000*
	**Vomiting**	151 (11.6%)	366 (5.5%)	0.000*	129 (5.1%)	0.000*
	**Belching**	135 (10.4%)	122 (1.8%)	0.000*	54 (2.1%)	0.000*
	**Abdominal pain**	81 (6.2%)	91 (1.4%)	0.000*	31 (1.2%)	0.000*
	**Constipation**	140 (10.8%)	146 (2.2%)	0.000*	64 (2.5%)	0.000*
	**Diarrhea**	219 (16.9%)	423 (6.4%)	0.000*	166 (6.6%)	0.000*
	**Abdominal distension/bloating**	108 (8.3%)	119 (1.8%)	0.000*	57 (2.3%)	0.000*
	**Other GI symptoms**	64 (4.9%)	53 (0.8%)	0.000*	18 (0.7%)	0.000*
	**Total GI symptoms**	813 (62.6%)	1656 (25.0%)	0.000*	682 (27.1%)	0.000*
	**GI symptoms as first**	123 (9.5%)	138 (2.1%)	0.000*	62 (2.5%)	0.000*
**Respiratory symptoms**	**Dry cough**	322 (24.8%)	2269 (34.2%)	0.000*	829 (32.9%)	0.000*
	**Productive cough**	791 (60.9%)	2757 (41.6%)	0.000*	1080 (42.9%)	0.000*
	**Sore throat**	848 (65.3%)	3612 (54.5%)	0.000*	1352 (53.7%)	0.000*
	**Nasal congestion/runny nose**	677 (52.2%)	2480 (37.4%)	0.000*	944 (37.5%)	0.000*
	**Dyspnea**	251 (19.3%)	674 (10.2%)	0.000*	279 (11.1%)	0.000*
	**Total respiratory symptoms**	1239 (95.5%)	5695 (85.9%)	0.000*	2179 (86.5%)	0.000*
**Fever**	**Fever degree**			0.000*		0.045*
	No (<37.2°C)	214 (16.5%)	1191 (18.0%)		453 (18.0%)	
	Low fever (≤39°C)	658 (50.7%)	3569 (53.8%)		1308 (51.9%)	
	High fever (>39°C)	413 (31.8%)	1718 (25.9%)	†	399 (15.8%)	†
**Severity**	**Pneumonia**	114 (8.8%)	170 (2.6%)	0.000*	85 (3.4%)	0.000*
	**Hospitalization**	83 (6.4%)	81 (1.2%)	0.000*	48 (1.9%)	0.000*
	**Course of infection (days)**	13.8±24.1	11.2±10.2	0.000*	11.4±10.2	0.000*

* The difference is statistically significant with the p-value less than 0.05;

^†^: Between-group comparisons by Bonferroni-corrected analyses showed significant differences in this subgroup (p-value < 0.05);

^¶^: The participants who experienced GI symptoms as the first symptoms of infection;

^§^: The P-value was obtained by comparing participants with preexisting digestive problems to a matched group derived from participants without preexisting digestive problems. In this study, 1,250 participants with preexisting digestive problems matched 2,519 control participants.

### The GI complaints during the omicron variant infection

In the study, 31.4% of the 7,929 participants reported GI symptoms. The most common GI symptoms were nausea (11.8%), followed by diarrhea (8.1%), vomiting (6.5%) and postprandial fullness (6.3%). More participants with preexisting digestive problems reported GI complaints than those without (62.6% vs 25.0%, p<0.001) (Table 1). The difference remained significant for all kinds of GI symptoms collected, including dysphagia, early satiety, postprandial fullness, nausea, vomiting, belching, abdominal pain, constipation, diarrhea, abdominal distension/bloating, and other GI complaints. After we eliminated the imbalance of the baseline general characteristics by propensity-score matching, all the above results remained (Table 1).

Besides, the GI symptoms during the omicron variant infection, including early satiety, postprandial fullness, nausea, vomiting, constipation, diarrhea, and abdominal distension/bloating, were more severe in participants with preexisting digestive problems (S2 Fig).

### Other clinical manifestations during the omicron variant infection

**Fig 2:**
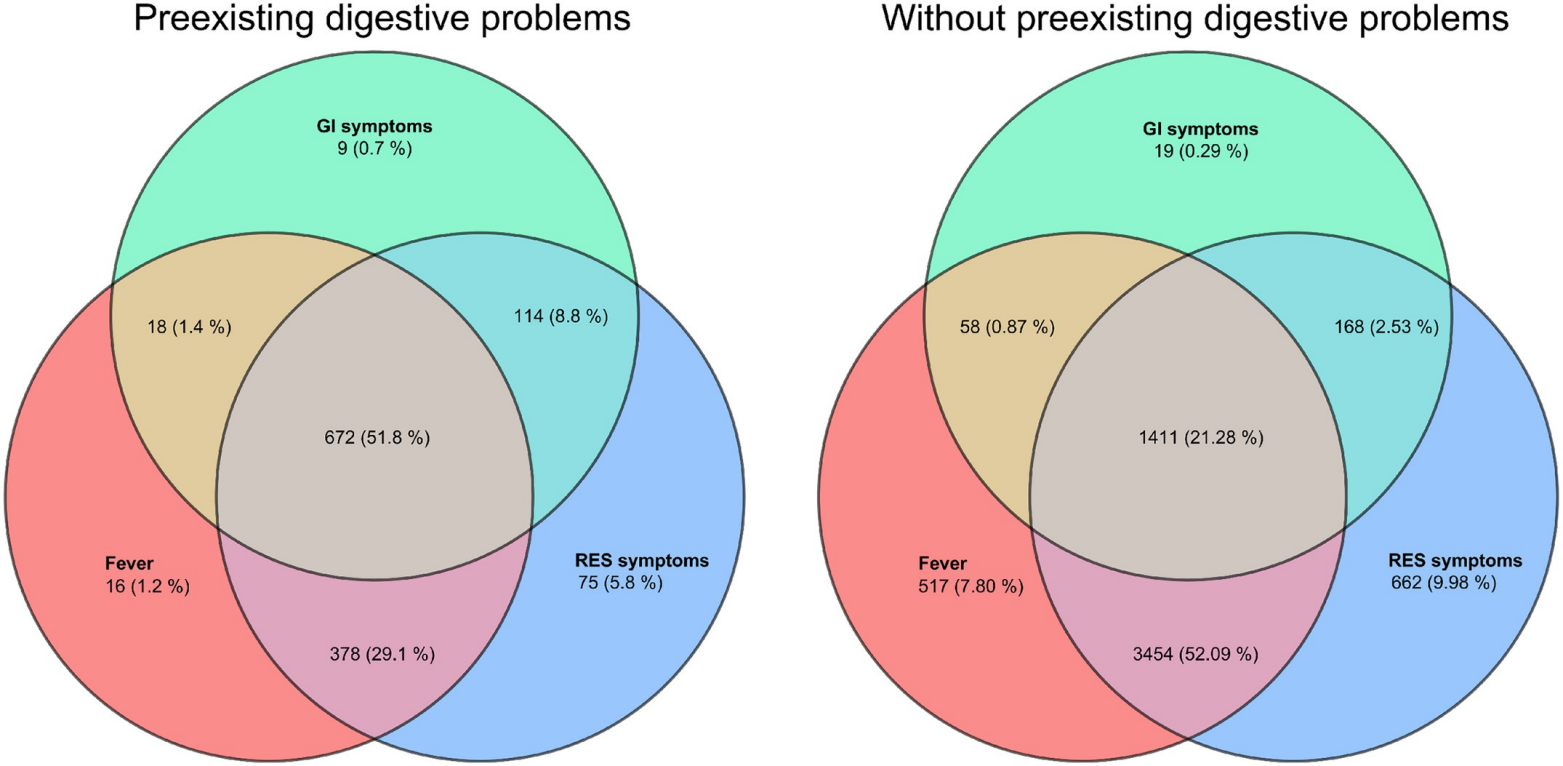
Venn diagram of the three different types of symptoms. RES symptoms donate respiratory symptoms. A Venn diagram consists of three overlapping circles, with each circle representing a kind of symptom (red for fever, green for GI symptoms; & blue for respiratory symptoms). The intersecting regions represent the intersection of those symptoms. And the number in each region represents the number of participants with those symptoms.

Participants with preexisting digestive problems experienced higher levels of fever and respiratory symptoms, such as dry cough, productive cough, sore throat, nasal congestion/runny nose, and difficulty breathing, compared to those with healthy digestive systems (Table 1 and Fig 2). Additionally, they were more likely to experience a combination of fever, respiratory symptoms, and gastrointestinal symptoms.

Additionally, a higher percentage of participants with preexisting digestive problems developed pneumonia (8.8% vs. 2.6%, p<0.001) and required hospitalization (6.4% vs. 1.2%, p<0.001) compared to those without. They also experienced a longer duration of infection. Even after we addressed the differences in the baseline general characteristics through propensity-score matching, the previously mentioned findings remained consistent (Table 1). We further categorized the participants with preexisting digestive problems into those with only GI diseases and with pre-existing symptoms, those with only preexisting GI symptoms and without GI diseases, and those with both pre-existing diseases and symptoms. All three groups of participants showed more severe clinical manifestations compared to matched control participants (S1 Table).

### The GI symptoms during infection were significantly associated with more severe clinical manifestations

There was a significantly higher proportion of females and a significantly lower proportion of smokers in participants with GI symptoms, regardless of the basic GI health status (Table 2). Besides, comorbidities were closely associated with GI symptoms in participants without preexisting digestive problems. Furthermore, the participants with GI symptoms reported significantly higher proportions of all the symptoms collected (respiratory symptoms and fever) compared to those without, except for fever in participants with preexisting digestive problems.

**Table 2:** Univariate analyses on participants with GI symptoms and those without based on their basic GI health status.

	Items	Preexisting digestive problems		P-value	No preexisting digestive problems		P-value
		With GI symptoms (N = 813)	Without GI symptoms (N = 485)		With new GI symptoms (N = 1,656)	Without GI symptoms (N = 4,975)	
**General characteristics**	**Sex (female)**	441 (54.2%)	228 (47.0%)	0.012*	962 (58.1%)	2594 (52.1%)	0.000*
	**Age (years old)**	36.9±9.4	36.9±10.6	0.654	35.8±8.6	35.8±8.6	0.909
	**BMI (kg/m** ^**2**^ **)**	23.5±3.8	23.7±3.7	0.092	23.2±3.6	23.4±3.6	0.162
	**Having smoking habit**	181 (22.3%)	135 (27.8%)	0.024*	256 (15.5%)	998 (20.1%)	0.000*
	**Alcohol consumption**	574 (70.6%)	320 (66.0%)	0.082	1008 (60.9%)	2998 (60.3%)	0.661
	**Prior COVID-19 infection**	45 (5.5%)	22 (4.5%)	0.431	32 (1.9%)	85 (1.7%)	0.549
	**Number of COVID-19 vaccinations**			0.672			0.256
	None	31 (3.8%)	14 (2.9%)		56 (3.4%)	139 (2.8%)	
	1–2	220 (27.1%)	131 (27.0%)		418 (25.2%)	1332 (26.8%)	
	≥3	562 (69.1%)	340 (70.1%)		1182 (71.4%)	3504 (70.4%)	
	**Use of NSAIDs**	605 (74.4%)	356 (73.4%)	0.740	1349 (81.5%)	3734 (75.1%)	0.000*
	**Comorbidities**	163 (20.1%)	79 (16.3%)	0.092	164 (9.9%)	393 (7.9%)	0.011*
**Respiratory symptoms**	**Dry cough**	186 (22.9%)	136 (28.0%)	0.037*	526 (31.8%)	1743 (35.0%)	0.015*
	**Productive cough**	525 (64.6%)	266 (54.9%)	0.001*	913 (55.1%)	1844 (37.1%)	0.000*
	**Sore throat**	552 (67.9%)	296 (61.0%)	0.012*	1100 (66.4%)	2512 (50.5%)	0.000*
	**Nasal congestion/runny nose**	475 (58.4%)	202 (41.7%)	0.000*	842 (50.9%)	1638 (32.9%)	0.000*
	**Dyspnea**	191 (23.5%)	60 (12.4%)	0.000*	311 (18.8%)	363 (7.3%)	0.000*
	**Total respiratory symptoms**	786 (96.7%)	453 (93.4%)	0.006*	1579 (95.4%)	4116 (82.7%)	0.000*
**Fever**	**Fever degree**			0.089			0.000*
	No (<37.2°C)	123 (15.1%)	91 (18.8%)		187 (11.3%)	1004 (20.2%)	†
	Low fever (≤39°C)	411 (50.6%)	247 (50.9%)		854 (51.6%)	2715 (54.6%)	†
	High fever (>39°C)	274 (33.7%)	139 (28.7%)		582 (35.1%)	1136 (22.8%)	†
**Severity**	**Pneumonia**	87 (10.7%)	27 (5.6%)	0.002*	65 (3.9%)	105 (2.1%)	0.000*
	**Hospitalization**	56 (6.9%)	27 (5.6%)	0.347	28 (1.7%)	53 (1.1%)	0.045*
	**Course of infection (days)**	12.9±11.8	13.3±11.8	0.468	11.7±9.6	11.0±10.4	0.000*

* The difference is statistically significant with the p-value less than 0.05;

^†^: Between-group comparisons by posthoc analyses (Bonferroni-corrected) showed significant differences in this subgroup (p-value < 0.05).

In terms of severity, a higher percentage of participants with GI symptoms developed pneumonia compared to those without (10.7% vs. 5.6%, p = 0.002 in participants with preexisting digestive problems; 3.9% vs. 2.1%, p<0.001 in participants without preexisting digestive problems). Participants without pre-existing digestive problems showed a significantly higher rate of hospitalization (1.7% vs. 1.1%, p = 0.045) and longer infection duration if they experienced new GI symptoms.

After propensity-score matching on general characteristics, the results changed slightly (S2 Table). The difference in hospitalization rates was no longer significant among participants who did not have preexisting digestive problems.

### Correlation between different GI symptoms and other clinical manifestations

**Fig 3:**
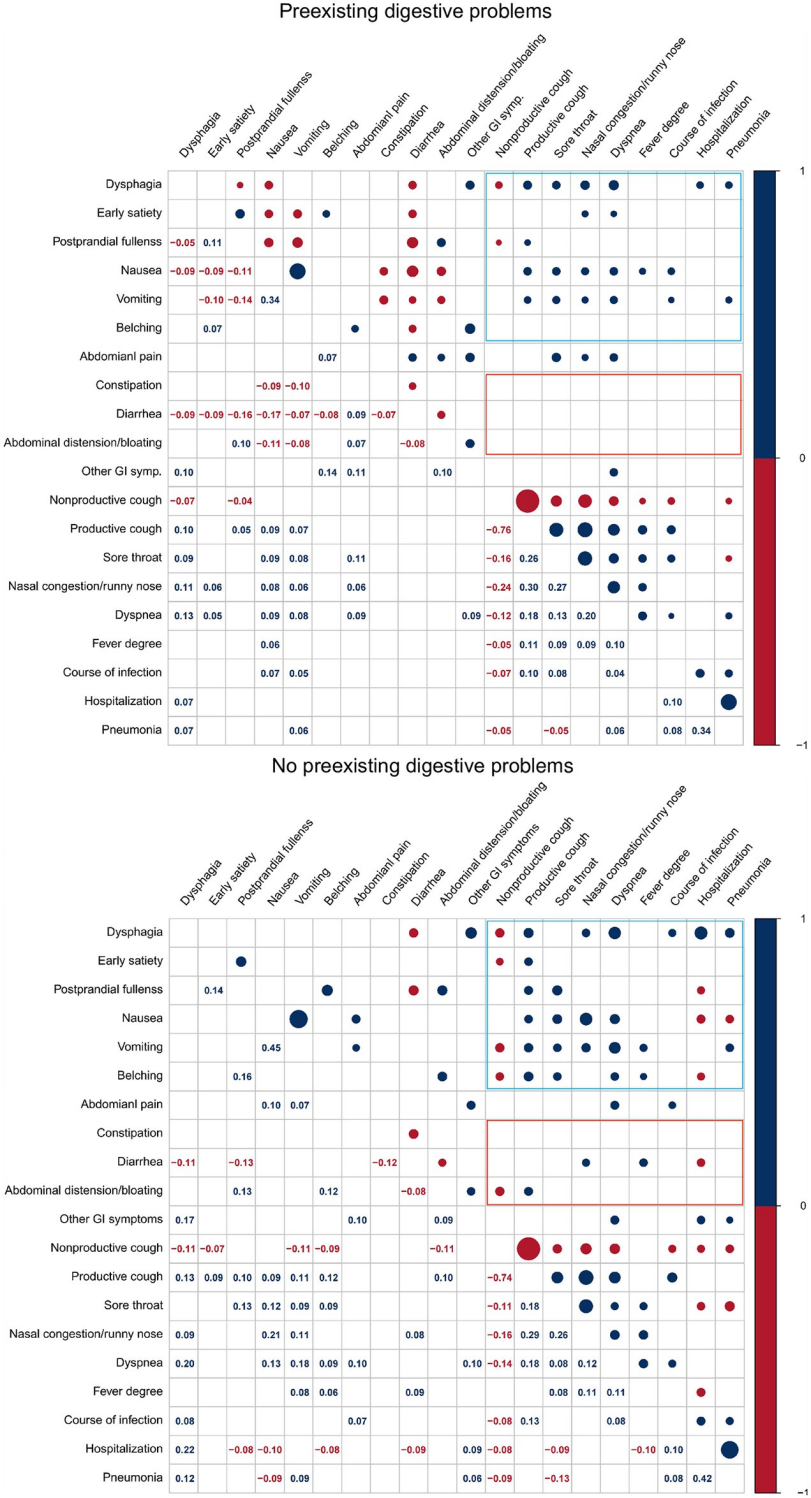
Correlation heatmaps between the GI symptoms and other clinical manifestations. The number showed the correlation coefficients, with the size of the dots matched the absolute value of the coefficients. Only significant correlations were displayed.

The above results found that the presence of GI symptoms was strongly correlated with other symptoms and clinical severity. However, this association may be different for different types of GI symptoms. We, therefore, further analyzed the correlation between the different symptoms and other symptoms and clinical severity (Fig 3). Upper gastrointestinal complaints (dysphagia, early satiety, postprandial fullness, nausea, vomiting, and belching), compared to lower gastrointestinal complaints (constipation, diarrhea, and abdominal distension/bloating), were more closely correlated with the incidence of respiratory symptoms and fever, the existing of pneumonia, hospitalization and course of infection, regardless of the GI health status.

### The associated factors of GI symptoms

Logistic regression was performed to find the associated factors of GI symptoms in all the participants. It was found that females, alcohol consumption, and comorbidities were associated factors in participants with preexisting digestive problems (Table 3). Interestingly, the smoking habit was significantly negatively associated with GI complaints. As for new-emerging GI symptoms in participants without preexisting digestive problems, the associated factors were the same in participants with preexisting digestive problems, except for the use of non-steroidal anti-inflammatory drugs (NSAIDs).

**Table 3:** Binary logistic regression for GI symptoms in the participants with preexisting digestive problems and new GI symptoms in the participants without preexisting digestive problems.

Independent variables	Preexisting digestive problems				No preexisting digestive problems			
	β	Wald	OR 95%CI	P-value	β	Wald	OR 95%CI	P-value
**Sex**	-0.27	4.00	0.76 (0.58, 0.99)	0.045*	-0.25	12.46	0.78 (0.68, 0.89)	0.000*
Male (vs. female)								
**Age (years old)**	-0.00	0.17	1.00 (0.99, 1.01)	0.679	0.00	0.00	1.00 (0.99, 1.00)	0.974
Continuous variable								
**BMI (kg/m** ^**2**^ **)**	-0.01	0.26	0.99 (0.96, 1.02)	0.608	-0.00	0.14	1.00 (0.98, 1.01)	0.704
Continuous variable								
**Smoking habit**	-0.35	5.08	0.71 (0.52, 0.96)	0.024*	-0.26	8.51	0.78 (0.65, 0.920	0.004*
With (vs. without)								
**Alcohol consumption**	0.39	8.51	1.47 (1.14, 1.91)	0.004*	0.18	8.14	1.20 (1.06, 1.37)	0.004*
With (vs. without)								
**Prior COVID-19 infection**	0.11	0.16	1.12 (0.65, 1.93)	0.690	0.20	0.89	1.22 (0.81, 1.85)	0.346
With (vs. without)								
**Number of COVID-19 vaccination**	-0.08	0.57	0.92 (0.74, 1.14)	0.452	0.02	0.09	1.02 (0.91, 1.13)	0.765
Rank variable ≥3 (vs. 1–2 vs. none)								
**Use of NSAIDs**	0.07	0.24	1.07 (0.82, 1.39)	0.626	0.39	29.38	1.48 (1.28, 1.70)	0.000*
Yes (vs. no)								
**Comorbidities**	0.33	4.07	1.40 (1.01, 1.93)	0.044*	0.33	10.17	1.40 (1.14, 1.71)	0.001*
With (vs. without)								

## Discussion

Our large-scale cross-sectional study investigated the GI symptoms and other clinical manifestations in Omicron infection based on the basic GI health status. In this study, we systematically evaluated the proportion of more than ten GI complaints (from upper GI to lower GI) in patients with omicron variant infection. We found that preexisting digestive problems were found to significantly influence the presence of GI symptoms during infection, while the presence of GI symptoms was strongly associated with more severe clinical manifestations.

In our study, the proportion of gastrointestinal (GI) symptoms in the general population at 31.1% (25.0% for participants without preexisting digestive problems) was higher than previous reports (17.6%, 95% CI: 12.3–24.5) for the early COVID-19 epidemic [3]. This may not be an indication that Omicron has a greater impact on the digestive system than previous variants. Because, many earlier studies did not evaluate GI symptoms other than diarrhea, nausea/vomiting, and abdominal pain [18]. On the contrary, a previous study reported the most common GI complaints were diarrhea (12.5%, 95%CI: 9.6–16.0), nausea/vomiting (10.2%, 95%CI: 6.6–15.3), and abdominal pain (9.2%, 95%CI: 5.7–14.5) with abdominal pain [3]. Thus, Omicron infection causes a lower proportion of many GI symptoms (diarrhea and abdominal pain) than prior variants. The omicron variant may have a lower impact on the GI tract compared with the early variant, consistent with previous reports suggesting a decreased virulence of the virus [19, 20].

As for the specific GI symptoms, we found the most common symptoms were nausea/vomiting (13.6%) and diarrhea (8.10%), while the incidence rate of abdominal pain was relatively low (2.2%). And the incidence of upper GI symptoms exceeded that of lower GI symptoms. In contrast, in previous studies, the incidence of lower gastrointestinal symptoms (diarrhea) tended to be the highest [3, 18, 21, 22] or comparable with the upper GI complaints (nausea and vomiting) [23, 24]. On the one hand, this suggests that the characteristics of GI symptoms caused by different variants are different, and Omicron is more likely to cause upper GI symptoms. On the other hand, our respondents had a very high rate of NSAIDs use, a class of medicines that can cause upper GI symptoms [25]. Our study also demonstrates that NSAIDs are indeed a risk factor for GI symptoms. Therefore, whether it is because the patients had more severe post-infectious symptoms that led them to choose to take the NSAIDs medication, thus partially contributing to the GI symptoms, needs to be further investigated. Caution needs to be exercised in the use of NSAIDs in patients with SARS-CoV-2 infections.

As for the relationship between basic GI health status and the incidence of GI symptoms, as well as the severity of the clinical manifestations, previous studies have shown conflicting results. A meta-analysis found that clinical manifestations of COVID-19 in IBD patients were similar to the general population [26]. Another meta-analysis found that in IBD patients affected by COVID-19, the risk ratio (RR) of adverse outcomes was increased compared to patients without IBD [27]. In our study, preexisting digestive problems were assessed not only by diagnosing various digestive system diseases but also by evaluating the gastrointestinal symptoms of the general public, offering a more comprehensive evaluation of their GI health status.

As for the relationship between GI complaints and the severity of infection, some previous studies were also inconsistent. For example, Menon *et al*. showed an increased risk of severe COVID-19 infection in the presence of GI symptoms [28]. A meta-analysis found that patients with GI complaints had a higher risk of acute respiratory distress syndrome [29]. However, other studies reported different results. A study reported no difference in the length of hospital stay between patients with GI and non-GI manifestations [30]. Another study suggested that patients with GI manifestations were less likely to develop severe-critical COVID-19 infection and had better outcomes [31]. Our work found the participants with GI symptoms during the infection tended to have a higher rate of high fever, pneumonia, and hospitalization and a longer course of infection The differences may be explained by distinct patient populations and different viral variants. Our study simultaneously examined the relationship between GI complaints and multiple dimensions of disease severity, and the conclusions were all consistent, so the results were relatively stable. This association was also consistent with the trend of basic GI health status and disease severity, suggesting the impact of GI involvement on infection at multiple levels.

Furthermore, in our work, upper GI symptoms were more closely correlated with other symptoms (respiratory symptoms and fever) and severity of infection than lower GI complaints. However, many previous studies have focused on the relationship between lower gastrointestinal complaints (such as diarrhea) and disease severity. Studies showed that diarrhea was strongly associated with severe infection with COVID-19 in the early epidemic [32, 33]. Meanwhile, few studies discussed upper GI complaints (such as vomiting and nausea). Therefore, more attention should be paid to patients’ upper GI complaints of Omicron infection.

We believe that assessing post-infectious gastrointestinal symptoms is clinically important: it can help healthcare professionals to accurately determine the condition of SARS-CoV-2-infected patients with gastrointestinal symptoms and identify patients with a poorer prognosis. Prevention of infections is even more important for people with pre-existing GI problems, as they are at risk of experiencing more severe GI injuries after contracting the virus. What’s more, several landmark studies have found that COVID-19 patients, particularly those who experienced reinfection, are at a higher risk of developing digestive diseases, including gastrointestinal tract and liver, gallbladder, and pancreas diseases [34–36]. It remains uncertain whether patients presenting with postinfectious gastrointestinal symptoms exhibit a higher propensity for developing these gastrointestinal disorders. Anyway, post-covid care should involve attention to gastrointestinal health and disease.

There are many limitations in our study. Firstly, this was a cross-sectional study. Accordingly, it was impossible to draw a causal relationship between the GI symptoms and the severity of clinical manifestations, as well as the use of NSAIDs. Secondly, although we used propensity matching and multiple logistic regression to reduce selection bias, the selection bias still existed because of the convenience sampling adopted. Thirdly, the nature of the participants’ GI symptoms, such as duration, symptom frequency, and symptom intensity, was not meticulously collected because it was difficult to get participants to fill out lengthy questionnaires. Fourthly, this is only a preliminary study, and many of the details were not explored in depth. We have thought about following up on those participants to explore GI damage after a long COVID-19 infection. However, after following up with a small sample of participants, we eventually gave up due to low compliance. Finally, we started to prepare for the study and apply for ethical review immediately after the epidemic outbreak, but this still led to a lag of more than two months in our study, which may lead to recall bias of the participants.

## Conclusion

Gastrointestinal symptoms were common in individuals infected with the omicron variant. Patients with gastrointestinal complaints during the omicron variant infection had the potential to experience more severe clinical manifestations. Basic gastrointestinal health status is closely related to the incidence of gastrointestinal complaints and the severity of the clinical manifestations.

## Supporting information

S1 Fig Venn diagram of the overlap of preexisting digestive illness and preexisting symptoms among the 1298 participants with preexisting digestive problems. (PNG)

S2 Fig The severity of gastrointestinal symptoms for participants with and without preexisting digestive problems. (PNG)

S1 Table Univariate analyses on the three sub-group participants with preexisting digestive problems with the matched control group, respectively. (DOCX)

S2 Table Univariate analyses on participants with new GI symptoms and those without based on their digestive health status after propensity-score matching. (DOCX)

S1 Data Raw questionnaire results with privacy information and some invalid questions that were not included in the analysis were removed. (XLSX)
