# Supplementary figures and images for: Impact of preexisting digestive problems on the gastrointestinal symptoms of patients with omicron variant of SARS-CoV-2 infection
Source: PLoS One. 2024 Oct 30;19(10):e0312545. doi: 10.1371/journal.pone.0312545 (PMC11524456; doi:10.1371/journal.pone.0312545)

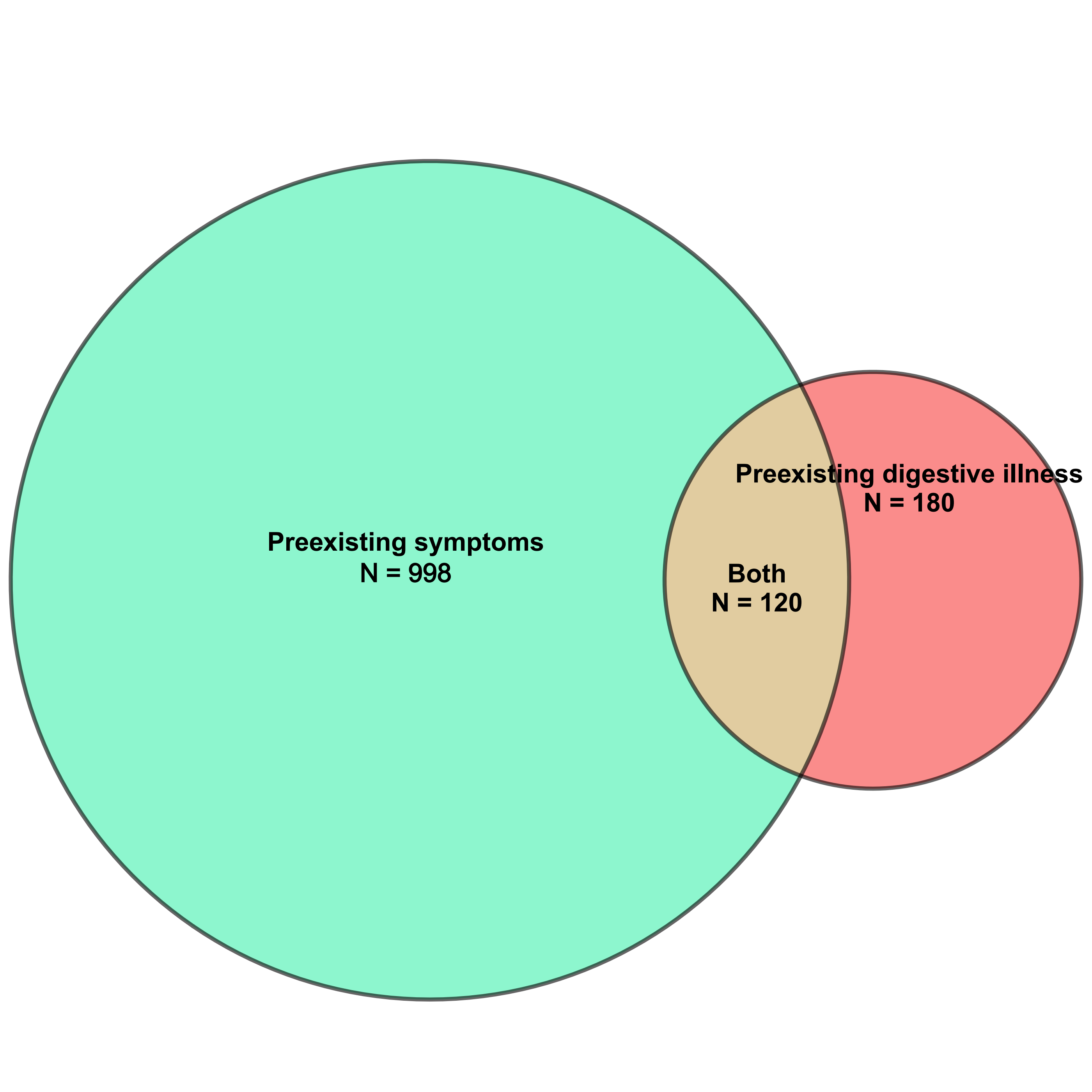

Supplement: S1 Fig — (PNG) [file pone.0312545.s001.png]
