# Supplementary material for: Impact of preexisting digestive problems on the gastrointestinal symptoms of patients with omicron variant of SARS-CoV-2 infection
Source: PLoS One. 2024 Oct 30;19(10):e0312545. doi: 10.1371/journal.pone.0312545 (PMC11524456; doi:10.1371/journal.pone.0312545)
Supplement: S1 Table — (DOCX) [file pone.0312545.s003.docx]

**S1 Table: Univariate analyses on the three sub-group participants with preexisting digestive problems with the matched control group, respectively**

|  | Items | Only preexisting digestive disease | Matched control | P-value | | Only preexisting GI symptoms | | Matched control | | P-value | | Both | | Matched control | | P-value | |
| --- | --- | --- | --- | --- | --- | --- | --- | --- | --- | --- | --- | --- | --- | --- | --- | --- | --- |
|  |  | N=168 | N=798 |  |  | N=991 | | N=3,882 | |  |  | N=102 | | N=459 | |  |  |
| General characteristics | **Gender (female%)** | 78 (46.4%) | 415 (52.0%) | | 0.189 | 521 (52.6%) | 2016 (51.9%) | | 0.718 | | 48 (47.1%) | | 212 (46.2%) | | 0.873 | |  |
|  | **Age (years)** | 37.8±10.7 | 37.4±9.5 | | 0.889 | 36.5±9.4 | 36.0±8.9 | | 0.194 | | 38.9±10.5 | | 37.0±9.4 | | 0.059 | |  |
|  | **BMI (kg/m^2^)** | 23.8±4.1 | 23.4±3.7 | | 0.263 | 23.6±3.7 | 23.5±3.7 | | 0.486 | | 23.4±3.7 | | 23.7±3.8 | | 0.431 | |  |
|  | **Having smoking habit** | 40 (23.8%) | 172 (21.6%) | | 0.521 | 225 (22.7%) | 867 (22.3%) | | 0.803 | | 28 (27.5%) | | 116 (25.3%) | | 0.649 | |  |
|  | **Alcohol consumption** | 97 (57.7%) | 487 (61.0%) | | 0.428 | 697 (70.3%) | 2721 (70.1%) | | 0.883 | | 69 (67.7%) | | 308 (67.1%) | | 0.916 | |  |
|  | **Prior COVID-19** | 38 (22.6%) | 152 (19.1%) | | 0.290 | 35 (3.5%) | 101 (2.6%) | | 0.113 | | 36 (35.3%) | | 133 (29.0%) | | 0.208 | |  |
|  | **Vaccination of COVID-19** |  |  | | 0.122 |  |  | | 0.614 | |  | |  | | 0.651 | |  |
|  | None | 12 (7.14%) | 29 (3.63%) | |  | 32 (3.2%) | 103 (2.7%) | |  | | 1 (1.0%) | | 11 (2.4%) | |  | |  |
|  | 1-2 | 44 (26.2%) | 217 (27.2%) | |  | 258 (26.0%) | 1020 (26.3%) | |  | | 31 (30.4%) | | 132 (28.8%) | |  | |  |
|  | ≥3 | 112 (66.7%) | 552 (69.2%) | |  | 701 (70.7%) | 2759 (71.1%) | |  | | 70 (68.6%) | | 316 (68.9%) | |  | |  |
|  | **Use of NSAIDs** | 123 (73.2%) | 601 (75.3%) | | 0.639 | 754 (76.1%) | 2944 (75.8%) | | 0.920 | | 74 (72.5%) | | 336 (73.2%) | | 0.888 | |  |
|  | **Comorbidities** | 38 (22.6%) | 152 (19.1%) | | 0.290 | 135 (13.6%) | 491 (12.7%) | | 0.413 | | 36 (35.3%) | | 133 (29.0%) | | 0.208 | |  |
| GI complaints during the infection | **Dysphagia** | 8 (4.8%) | 12 (1.5%) | | 0.007* | 90 (9.1%) | 85 (2.2%) | | 0.000* | | 12 (11.8%) | | 4 (0.9%) | | 0.000* | |  |
|  | **Early satiety** | 6 (3.6%) | 26 (3.3%) | | 0.837 | 96 (9.7%) | 122 (3.1%) | | 0.000* | | 10 (9.8%) | | 15 (3.3%) | | 0.004* | |  |
|  | **Postprandial fullness** | 11 (6.6%) | 38 (4.8%) | | 0.338 | 179 (18.1%) | 180 (4.6%) | | 0.000* | | 22 (21.6%) | | 17 (3.7%) | | 0.000* | |  |
|  | **Nausea** | 15 (8.9%) | 69 (8.7%) | | 0.906 | 224 (22.6%) | 393 (10.1%) | | 0.000* | | 27 (26.5%) | | 50 (10.9%) | | 0.000* | |  |
|  | **Vomiting** | 10 (6.0%) | 37 (4.6%) | | 0.471 | 121 (12.2%) | 196 (5.1%) | | 0.000* | | 15 (14.7%) | | 19 (4.1%) | | 0.000* | |  |
|  | **Belching** | 4 (2.4%) | 12 (1.5%) | | 0.418 | 107 (10.8%) | 84 (2.2%) | | 0.000* | | 20 (19.6%) | | 7 (1.5%) | | 0.000* | |  |
|  | **Abdominal pain** | 6 (3.6%) | 13 (1.6%) | | 0.099 | 57 (5.8%) | 46 (1.2%) | | 0.000* | | 16 (15.7%) | | 5 (1.1%) | | 0.000* | |  |
|  | **Constipation** | 2 (1.2%) | 14 (1.8%) | | 0.603 | 125 (12.6%) | 98 (2.5%) | | 0.000* | | 10 (9.8%) | | 14 (3.1%) | | 0.002* | |  |
|  | **Diarrhea** | 16 (9.5%) | 41 (5.1%) | | 0.028* | 170 (17.2%) | 256 (6.6%) | | 0.000* | | 30 (29.4%) | | 30 (6.5%) | | 0.000* | |  |
|  | **Abdominal distension/bloating** | 3 (1.8%) | 10 (1.3%) | | 0.586 | 85 (8.6%) | 72 (1.9%) | | 0.000* | | 19 (18.6%) | | 6 (1.3%) | | 0.000* | |  |
|  | **Other GI symptoms** | 1 (0.6%) | 2 (0.3%) | | 0.466 | 54 (5.5%) | 28 (0.7%) | | 0.000* | | 8 (7.8%) | | 1 (0.2%) | | 0.000* | |  |
|  | **Total GI symptoms** | 57 (33.9%) | 182 (22.8%) | | 0.002* | 641 (64.7%) | 1022 (26.3%) | | 0.000* | | 87 (85.3%) | | 114 (24.8%) | | 0.000* | |  |
|  | **GI symptoms as first**¶ | 12 (7.1%) | 16 (2.0%) | | 0.000* | 907 (91.5%) | 3796 (97.8%) | | 0.000* | | 17 (16.7%) | | 11 (2.4%) | | 0.000* | |  |
| Respiratory symptoms | **Dry cough** | 46 (27.4%) | 277 (34.7%) | | 0.067 | 248 (25.0%) | 1325 (34.1%) | | 0.000* | | 22 (21.6%) | | 163 (35.5%) | | 0.007* | |  |
|  | **Productive cough** | 91 (54.2%) | 314 (39.4%) | | 0.000* | 616 (62.2%) | 1639 (42.2%) | | 0.000* | | 64 (62.8%) | | 174 (37.9%) | | 0.000* | |  |
|  | **Sore throat** | 81 (48.2%) | 420 (52.6%) | | 0.298 | 688 (69.4%) | 2111 (54.4%) | | 0.000* | | 68 (66.7%) | | 230 (50.1%) | | 0.002* | |  |
|  | **Nasal congestion/runny nose** | 78 (46.4%) | 282 (35.3%) | | 0.007* | 526 (53.1%) | 1470 (37.9%) | | 0.000* | | 56 (54.9%) | | 169 (36.8%) | | 0.001* | |  |
|  | **Dyspnea** | 23 (13.7%) | 77 (9.7%) | | 0.118 | 205 (20.7%) | 410 (10.6%) | | 0.000* | | 16 (15.7%) | | 45 (9.8%) | | 0.084 | |  |
|  | **Total respiratory symptoms** | 153 (91.1%) | 679 (85.1%) | | 0.041* | 952 (96.1%) | 3362 (86.6%) | | 0.000* | | 99 (97.1%) | | 390 (85.0%) | | 0.000* | |  |
| Fever | **Fever** |  |  | | 0.810 |  |  | | 0.000* | |  | |  | | 0.168 | |  |
|  | No (<37.2℃) | 36 (21.4%) | 151 (18.9%) | |  | 148 (14.9%) | 690 (17.8%) | | † | | 14 (13.7%) | | 98 (21.4%) | |  | |  |
|  | Low fever (≤39℃) | 92 (54.8%) | 443 (55.5%) | |  | 503 (50.8%) | 2061 (53.1%) | |  | | 56 (54.9%) | | 236 (51.4%) | |  | |  |
|  | High fever (>39℃) | 39 (23.2%) | 186 (23.3%) | |  | 329 (33.2%) | 1043 (26.9%) | | † | | 31 (30.4%) | | 114 (24.8%) | |  | |  |
| Severity | **Pneumonia** | 16 (9.5%) | 22 (2.8%) | | 0.000* | 56 (5.7%) | 115 (3.0%) | | 0.000* | | 19 (18.6%) | | 22 (4.8%) | | 0.000* | |  |
|  | **Hospitalization** | 19 (11.3%) | 10 (1.3%) | |  | 30 (3.0%) | 53 (1.4%) | |  | | 12 (11.8%) | | 10 (2.2%) | |  | |  |
|  | **Course of infection (days)** | 13.5±12.4 | 11.3±11.0 | | 0.002* | 13.5±26.2 | 11.2±9.7 | | 0.000* | | 16.0±16.5 | | 11.5±10.2 | | 0.000* | |  |

* The difference is statistically significant with the p-value less than 0.05;

†: Between-group comparisons by Bonferroni-corrected analyses showed significant differences in this subgroup (p-value < 0.05).
