# Supplementary material for: Impact of preexisting digestive problems on the gastrointestinal symptoms of patients with omicron variant of SARS-CoV-2 infection
Source: PLoS One. 2024 Oct 30;19(10):e0312545. doi: 10.1371/journal.pone.0312545 (PMC11524456; doi:10.1371/journal.pone.0312545)
Supplement: S2 Table — (DOCX) [file pone.0312545.s004.docx]

**S2 Table: Univariate analyses on participants with new GI symptoms and those without based on their digestive health status after propensity-score matching**

|  | Items | Preexisting digestive illness | | P-value | No preexisting digestive illness | | P-value |
| --- | --- | --- | --- | --- | --- | --- | --- |
|  |  | With GI symptoms (N=476) | Without GI symptoms (N=476) |  | With GI symptoms (N=1,654) | Without GI symptoms (N=3,285) |  |
| General characteristics | **Sex (female)** | 225 (47.3%) | 225 (47.3%) | 1.000 | 962 (58.2%) | 1904 (58.0%) | 0.892 |
|  | **Age (years)** | 35.8±8.8 | 36.9±10.6 | 0.246 | 35.8±8.6 | 35.8±8.8 | 0.977 |
|  | **BMI (kg/m^2^)** | 23.8±4.0 | 23.7±3.7 | 0.791 | 23.2±3.6 | 23.2±3.6 | 0.966 |
|  | **Having smoking habits** | 130 (27.3%) | 130 (27.3%) | 1.000 | 255 (15.4%) | 509 (30.8%) | 0.943 |
|  | **Alcohol consumption** | 318 (66.8%) | 318 (66.8%) | 1.000 | 1007 (60.9%) | 1993 (60.7%) | 0.885 |
|  | **Prior COVID-19 infection** | 19 (4.0%) | 19 (4.0%) | 1.000 | 30 (1.8%) | 55 (1.7%) | 0.722 |
|  | **Number of COVID-19 vaccinations** |  |  | 0.099 |  |  | 0.333 |
|  | None | 22 (4.6%) | 13 (2.7%) |  | 56 (3.4%) | 87 (2.7%) |  |
|  | 1-2 | 147 (30.9%) | 129 (27.1%) |  | 417 (25.2%) | 846 (25.8%) |  |
|  | ≥3 | 307 (64.5%) | 334 (70.2%) |  | 1181 (71.4%) | 2352 (71.6%) |  |
|  | **Use of NSAIDs** | 349 (73.3%) | 349 (73.3%) | 1.000 | 1347 (81.4%) | 2423 (73.8%) | 0.000* |
|  | **Comorbidities** | 76 (16.0%) | 76 (16.0%) | 1.000 | 163 (9.9%) | 305 (9.3%) | 0.518 |
| Respiratory | **Dry cough** | 110 (23.1%) | 133 (27.9%) | 0.087 | 526 (31.8%) | 1124 (34.2%) | 0.090 |
| symptoms | **Productive cough** | 296 (62.2%) | 264 (55.1%) | 0.035* | 911 (55.1%) | 1275 (38.8%) | 0.000* |
|  | **Sore throat** | 316 (66.4%) | 294 (61.8%) | 0.137 | 1098 (66.4%) | 1677 (51.1%) | 0.000* |
|  | **Nasal congestion/runny nose** | 275 (57.8%) | 198 (41.6%) | 0.000* | 841 (50.9%) | 1106 (33.7%) | 0.000* |
|  | **Dyspnea** | 123 (25.8%) | 59 (12.4%) | 0.000* | 310 (18.7%) | 261 (8.0%) | 0.000* |
|  | **Total respiratory symptoms** | 457 (96.0%) | 447 (95.7%) | 0.139 | 1577 (95.3%) | 2740 (83.4%) | 0.000* |
| Fever | **Fever degree** |  |  | 0.459 |  |  | 0.000* |
|  | No (<37.2℃) | 78 (16.4%) | 86 (18.1%) |  | 187 (11.3%) | 679 (20.7%) | † |
|  | Low fever (≤39℃) | 239 (50.2%) | 245 (51.5%) |  | 854 (51.6%) | 1773 (54.0%) |  |
|  | High fever (>39℃) | 155 (32.6%) | 137 (28.8%) |  | 580 (35.1%) | 759 (23.1%) | † |
| Severity | **Pneumonia** | 49 (10.3%) | 27 (5.7%) | 0.009* | 65 (3.9%) | 79 (2.4%) | 0.003* |
|  | **Hospitalization** | 34 (7.1%) | 25 (5.3%) | 0. | 28 (1.7%) | 38 (1.2%) | 0.121 |
|  | **Course of infection (days)** | 12.8±11.2 | 12.8±11.1 | 0.705 | 11.6±9.6 | 11.0±10.1 | 0.000* |

* The difference is statistically significant with the p-value less than 0.05;

†: Between-group comparisons by Bonferroni-corrected analyses showed significant differences in this subgroup (p-value < 0.05).
